# Extending classic Haddon’s matrix for road safety promotion: toward better applicability 

**Published:** 2019-08

**Authors:** Naser Derakhshani, Homayoun Sadeghi-Bazarghani, Mahdiyeh Heydari, Ramin Rezapour, Hojat Gharaee

**Affiliations:** ^*a*^Student Research Committee, Iran University of Medical Sciences, Tehran, Iran.; ^*b*^Road Traffic Injury Research Center, Tabriz University of Medical Sciences, Tabriz, Iran.; ^*c*^Student Research Committee, Tehran University of Medical Sciences, Tehran, Iran.; ^*d*^Candidate in Health Services Management, Tabriz Health Services Management Research Center, Health Management and Safety Promotion Research Institute, Tabriz University of Medical Sciences, Tabriz, Iran.

## Abstract

**Background:**

Various models have been used to portray the causal pattern of Road Traffic Injuries (RTIs). Of which one most frequently used is the Haddon’s matrix proposed in 1970. The aim of this study was to improve applicability of this model through extending for road safety promotion.

**Methods:**

Required data gathered from 57 articles on influencing factors in RTIs and analyzed using Content- Analysis method by using the Haddon’s matrix. Six national expert’s opinions were applied in extension of model.

**Results:**

Initially 467 factors were extracted. After removing the duplicates and merging the similar factors, finally 76 factors remained. The pre-event phase had the most factors (N=41). Of the columns, the Vehicle and equipment had the most factors (N=26). According to extracted factors and experts opinion columns extended to 7 categories (human, environmental, vehicle and equipment, road, socio-economic, structure and organizational, mixed). Rows extended to two categories included: pre-event (long term and short term) and post-event (crash scene, transfer, care delivery).

**Conclusions:**

In this study the results of the studies on influencing factors in RTIs and the experts’ opinions were used to develop an expanded model of Haddon’s Matrix which is specific to traffic accidents. Future studies might investigate the effectiveness of this expanded model.

**Keywords:**

Prevention, Road Traffic Injuries, Haddon matrix, Causal pattern, applicability

